# Six degrees of freedom CBCT‐based positioning for intracranial targets treated with frameless stereotactic radiosurgery

**DOI:** 10.1120/jacmp.v13i6.3916

**Published:** 2012-11-08

**Authors:** Anees Dhabaan, Eduard Schreibmann, Arsalan Siddiqi, Eric Elder, Tim Fox, Tomi Ogunleye, Natia Esiashvili, Walter Curran, Ian Crocker, Hui‐Kuo Shu

**Affiliations:** ^1^ Department of Radiation Oncology and Winship Cancer Institute Emory University Atlanta GA USA

**Keywords:** stereotactic frameless radiosurgery, six degrees of freedom, registration, cone beam CT

## Abstract

Frameless radiosurgery is an attractive alternative to the framed procedure if it can be performed with comparable precision in a reasonable time frame. Here, we present a positioning approach for frameless radiosurgery based on in‐room volumetric imaging coupled with an advanced six‐degrees‐of‐freedom (6 DOF) image registration technique which avoids use of a bite block. Patient motion is restricted with a custom thermoplastic mask. Accurate positioning is achieved by registering a cone‐beam CT to the planning CT scan and applying all translational and rotational shifts using a custom couch mount. System accuracy was initially verified on an anthropomorphic phantom. Isocenters of delineated targets in the phantom were computed and aligned by our system with an average accuracy of 0.2 mm, 0.3 mm, and 0.4 mm in the lateral, vertical, and longitudinal directions, respectively. The accuracy in the rotational directions was 0.1°, 0.2°, and 0.1° in the pitch, roll, and yaw, respectively. An additional test was performed using the phantom in which known shifts were introduced. Misalignments up to 10 mm and 3° in all directions/rotations were introduced in our phantom and recovered to an ideal alignment within 0.2 mm, 0.3 mm, and 0.4 mm in the lateral, vertical, and longitudinal directions, respectively, and within 0.3° in any rotational axis. These values are less than couch motion precision. Our first 28 patients with 38 targets treated over 63 fractions are analyzed in the patient positioning phase of the study. Mean error in the shifts predicted by the system were less than 0.5 mm in any translational direction and less than 0.3° in any rotation, as assessed by a confirmation CBCT scan. We conclude that accurate and efficient frameless radiosurgery positioning is achievable without the need for a bite block by using our 6 DOF registration method. This system is inexpensive compared to a couch‐based 6 DOF system, improves patient comfort compared to systems that utilize a bite block, and is ideal for the treatment of pediatric patients with or without general anesthesia, as well as of patients with dental issues. From this study, it is clear that only adjusting for 4 DOF may, in some cases, lead to significant compromise in PTV coverage. Since performing the additional match with 6 DOF in our registration system only adds a relatively short amount of time to the overall process, we advocate making the precise match in all cases.

PACS number: 87.55.tm; 87.55.Qr; 87.57.nj

## I. INTRODUCTION

Stereotactic radiosurgery (SRS) is routinely performed since the advent of the Gamma Knife radiosurgery system (Elekta, Stockholm, Sweden) developed in 1968. SRS is performed with the use of a stereotactic head ring rigidly affixed to the patient's skull to provide rigid patient immobilization during simulation and treatment delivery. Headframe‐based radiosurgery has been shown to provide excellent target positioning during treatment delivery.^1^, ^2^

Frame‐based SRS provides a high degree of accuracy, which is necessary when using a large and highly conformal radiation dose, and has been considered the gold standard for radiosurgery. However, it is an invasive and painful system that is not suitable for every patient. Situations where this type of system may present problems include the immobilization of young children and infants who have a thin skull that may not tolerate the torque generated by the fixation pin, and adult patients with a previous craniotomy bone flap that cannot have a head pin placed into it. Various noninvasive frameless SRS systems have been evaluated. For example, a noninvasive frame (Laitinen frame) made of reinforced plastic and aluminum that is mounted into the patient's head by means of two earplugs and a nasal support has been used and its accuracy has been evaluated.^(3)^ While this system was used for both children and adults, it still produced some discomfort, or pain in the ear and nose during treatments. In addition, this system has only been used for fractionated stereotactic radiotherapy (FSRT). Other methods of target localization include the use of a fusion of computed tomography and linear accelerator (FOCAL) unit that utilized the CT for target positioning and immobilized the cranium by using a conventional heat‐flexible head mold,^4^, ^5^ and the use of Mobile CT with mask immobilization.^(6)^ These CT‐guided systems were superior in accuracy compared to other systems, and were made feasible by great technological advances in the field through the availability of image‐guided systems with most linear accelerators.

Recently, linear accelerators have begun to be equipped with imaging systems that permit image‐guided alignment for radiotherapy. With these advances, interest has increased in using image guidance for alignment with nonrigid immobilization such as thermoplastic mask. In such cases, less invasive systems were used that did not require a fixed headframe.^(7)^ One such system involved the use of a thermoplastic mask in conjunction with an optically‐guided bite plate for immobilization and targeting. Target positioning accuracy of this optically‐guided system for linear accelerator‐based frameless SRS has been evaluated and was found to provide a localization accuracy of 1.1±0.3  mm relative to the head ring system.^7^, ^12^

At our institution, a linear accelerator‐based framed stereotactic radiosurgery program was first established in 1989 with introduction of a frameless stereotactic alternative in 2003. This frameless system employed an optical tracking system with a bite plate attached to the patient's maxillary dentition. Although the bite plate optically‐guided system accuracy was found to be similar to what has been previously reported, in our practice, this system was always used in conjunction with cone‐beam CTs (CBCTs) acquired using our linear accelerator's on‐board imaging system. When the frameless program was first implemented, CBCTs were used mainly to confirm localization achieved with optical guidance. However, this imaging did reveal instances where issues with the bite block fit was noted, leading to significant degradation of positioning accuracy. In addition, optical guidance was used to monitor patient motion during treatment and repositioning between treatment arcs when patient movement exceeded tolerance. While this monitoring provided reassurance with regard to patient immobilization, our experience suggested that intrafraction movement was not a significant issue. The greatest limitation of the bite block‐based frameless system was that it was not suitable for many elderly patients who frequently lack maxillary teeth, nor for young children who could not tolerate this mouthpiece over the time needed for treatment. Given the limitations of this system including the need for full patient cooperation and the reproducibility of bite plate reseating, we explored alternatives for patient localization. Therefore, in 2009, we developed a frameless radiosurgery system that no longer required a bite plate, and was suitable for single and multiple fractions high‐dose treatment with radiation.

This study presents a positioning approach for frameless radiosurgery treatment based on in‐room volumetric imaging coupled with an advanced six‐degrees‐of‐freedom (6 DOF) image registration technique. Avoiding use of a hard‐frame ring or a bite plate makes this system suitable for a wider variety of patients. Many studies have investigated the use of planar imaging systems to guide frameless SRS/FSRT treatments.^9^, ^13^, ^15^ Several studies have shown that anatomical structures and soft tissues are better visualized on axial images of CBCT than planar images.^14^, ^16^, ^18^ We therefore designed our system to implement use of kV‐CBCT, in addition to planar images, to guide frameless SRS/FSRT treatment utilizing our 6 DOF registration system.

Accurate positioning is achieved in this study by matching an in‐room CBCT to the planning CT (PCT) using a 6 DOF rigid registration method utilizing the image registration software VelocityAI. This registration system calculates shifts along three angles (rotations around left–right direction (pitch or tilt), superior–inferior direction (roll or spin), and anterior–posterior direction (yaw)) and three directions (translational shifts along left–right (lateral), anterior–superior (vertical), and superior–inferior (longitudinal) axes). All of these setup parameters are calculated after acquiring the CBCT and immediately applied to correct patient setup. All translational and yaw rotational shifts are compared to their corresponding values predicated by the Varian 4D Online Review software.

## II. MATERIALS AND METHODS

### A. Frameless radiosurgery treatment procedure

All frameless SRS/FSRT procedures performed at our institution are treated using a Novalis TX linear accelerator (Varian Medical Systems, Palo Alto, CA). This unit is equipped with a high‐definition multi‐leaf collimator (MLC, 2.5 mm leaf width at the isocenter) and a kV onboard imaging system (OBI). The OBI system consists of a kV X‐ray source and a flat‐panel amorphous silicon (aSi) detector mounted onto the gantry of the linear accelerator via controlled arms in an orthogonal direction with respect to the MV beam. The CBCT is acquired by rotating the gantry around the patient while the kV source generates X‐rays covering an area 50 cm wide by 17 cm long. The axial images are reconstructed after the projection data are acquired. The CBCT data are saved and exported automatically into Velocity AI (Velocity Medical Systems, Atlanta, GA).

**Figure 1:**
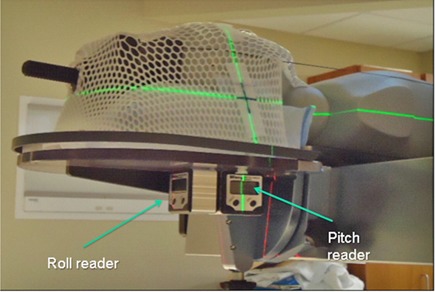
Customized couch mount showing the locations of pitch and roll digital readers.

A custom thermoplastic mask restricts patient motion during setup and treatment. Accurate positioning is achieved by matching an in‐room CBCT to the PCT using a 6 DOF rigid registration method customized to use mutual information metric in Mattes's formulation.^(19)^ This registration system online calculates shifts along three angles [rotations around left–right direction (pitch or tilt), superior–inferior direction (roll or spin), and anterior–posterior direction (yaw)) and three directions (translational shifts along left–right (lateral), anterior–superior (vertical), and superior–inferior (longitudinal) axes). The spin and tilt are applied using a customized couch mount (Fig. 1), while couch translational and rotational (yaw) shifts are applied using the treatment console. The rotational accuracy of this customized couch mount is 0.1° and the couch used has a translational accuracy of 1 mm. In addition to our 6 DOF system, the Varian 4D Online Review System (Varian Medical Systems, Palo Alto, CA) was used for clinical verification of patient positioning.

### B. Phantom study

Prior to using our 6 DOF system for patient setup, the accuracy of the system was evaluated using an anthropomorphic phantom. Three cylindrical inserts of high density materials were inserted in three locations in the head of the CIRS (Computerized Imaging Reference Systems, Inc., Norfolk, VA) pediatric anthropomorphic phantom. The phantom had bone, brain, soft tissues, lung, and air volumes with shapes and attenuation characteristics simulating a pediatric patient anatomy.^(20)^ A headrest and a U‐frame custom thermoplastic mask (WFR/Aquaplast Corp., Wyckoff, NJ) were fabricated to fix the phantom onto the head restraint base, as shown in Fig. 1.

A CT scan of the phantom was obtained at 0.625 mm slice thickness using a GE Lightspeed 16 multislice spiral CT scanner (General Electric Medical Systems, Waukesha, WI). Eclipse Treatment Planning System (Varian Medical Systems, Palo Alto, CA) was used to create a treatment plan. The cylindrical inserts were contoured to create target isocenters. For each target's isocenter, the other two inserts were used to visualize the accuracy of the fusion between the CBCT and the PCT.

Prior to positioning the phantom onto the linear accelerator table, a 10 mm stereotactic cone was secured to the Novalis TX. The isocenter was determined by reviewing the projection of light field on a spherical marker with gantry and couch rotation. The Winston‐Lutz^(21)^ test was then conducted to confirm the alignment by comparing the marker position at the linear accelerator isocenter to the marker portal images of various combination of gantry and couch angles obtained with the stereotactic cone. Verification of the image guidance isocenters was performed using X‐ray alignment and image guidance isocenter calibration phantom. The lasers were then aligned to the linear accelerator isocenter. Hence, the best isocenter was determined.

**Figure 2:**
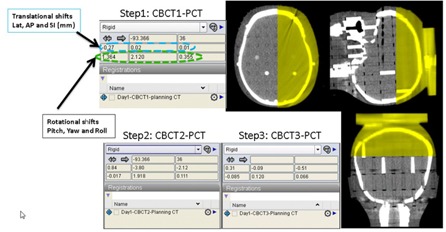
Pediatric phantom setup using our 6 DOF system. The planning CT is in gray scale and the final verification CBCT is in color.

For phantom target alignment, the phantom was initially setup using the laser marks on the mask. Then two orthogonal kV images were taken and registered using their corresponding digitally reconstructed radiographs (DRR) utilizing Varian's 3D Online Review Software. The calculated three translational shifts were applied. Next, a CBCT was acquired and registered to the PCT utilizing the 6 DOF registration system. While this system gives three translational and three angular rotational shifts (Fig. 2), at this step, only the spin and tilt were applied by dialing in the rotational adjustments in the couch mount. Then, a second CBCT was obtained and again fused to the PCT using the 6 DOF registration, and all translational and the couch rotational (yaw) shifts were applied (the spin and tilt which was previously adjusted should not require further adjustment). A third CBCT was obtained to verify the residual setup errors again using 6 DOF registration. To evaluate interfraction setup variability, the phantom setup was repeated five different times and each residual setup error calculated. In every case, the CBCT was used to find the isocenters.

To further validate the system's accuracy beyond the best match between the third CBCT and the PCT for the phantom, we introduced a known translational and rotational misalignment. Then a fourth CBCT was taken and registered with the PCT using our 6 DOF system. The phantom isocenters was intentionally shifted ± 5 mm and ± 10 mm in lateral, vertical, and longitudinal directions, while keeping the angular coordinates constant. Similarly, angular shifts of 1°, 2°, and 3° were introduced in both clockwise and counterclockwise directions, while keeping translational coordinates constant. After each shift was introduced, a CBCT was taken and registered to the PCT using our 6 DOF alignment system.

### C. Patient study

The first 28 patients treated at our institution using this frameless system were positioned in a similar manner to the phantom described previously. These patients had 38 targets and a total of 63 isocenters. All patients were immobilized using a customized headrest and U‐frame mask. Head CT scans were acquired with 0.625 mm slice thickness without localizer and fused to their MRIs. Treatment plans were created using the Eclipse treatment planning system.

**Figure 3:**
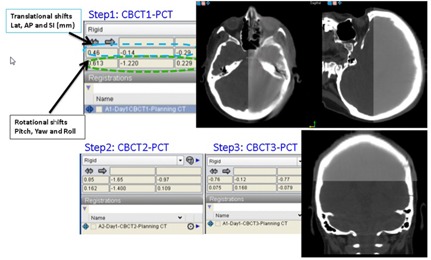
Representative clinical case: dark gray scale is the planning CT and the light gray is final the verification CBCT.

Initially, patients were aligned using the marked isocenter. The spin and tilt angle readers were reset to zero. Then, an AP and lateral kV images were taken and registered to the corresponding DRRs. The calculated translational couch shifts were applied. A CBCT is then acquired and registered to the PCT using the 6 DOF system (Fig. 3). The required spin and tilt rotational adjustments were made. Then, a second CBCT was obtained and again registered to the PCT using the 6 DOF system. Now, the calculated three translational and couch rotational (yaw) shifts were subsequently applied prior to patient treatment delivery. With every CBCT and PCT registration, the Varian 4D online review system was used to verify the patient positioning as determined by the 6 DOF system.

**Figure 4:**
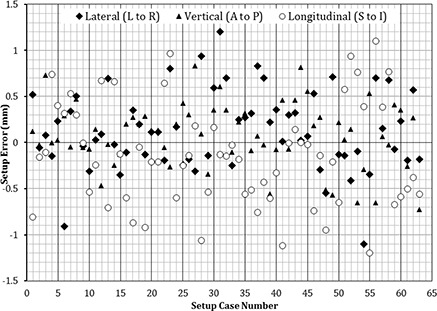
Translational residual setup errors calculated for all frameless clinical treatment fractions using 6 DOF system.

**Figure 5:**
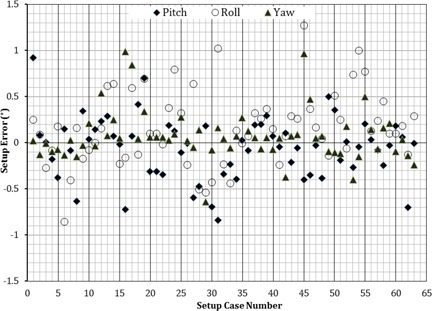
Rotational residual setup errors calculated for all frameless clinical treatment fractions using 6 DOF system.

For each patient in this study, a third CBCT was taken to verify the accuracy of the calculated shifts prior to treatment delivery. However, after analyzing our data, we concluded that the third CBCT was not necessary because only negligible rotational and translational shifts were noted (Figs. 4 and 5). Finally, in this study, a retrospective analysis was conducted for all frameless SRS/FSRT treatment setups performed using our 6 DOF system to evaluate the residual setup errors and their magnitude.

## III. RESULTS

Our phantom study showed that our 6 DOF alignment system positions the target's isocenter within 0.4 mm in any translational and within 0.2° in any rotational direction, as shown in Table 1. Additionally, to test the system's limitations, known misalignments were introduced after achieving best alignment, which was confirmed by the 6 DOF system registration of a third CBCT to the PCT. Misalignments up to 10 mm in translational and 3° rotational shifts were introduced and a fourth CBCT was obtained and registered to the PCT. As shown in Table 1, these misalignments were recovered to within an average accuracy of 0.2 mm in lateral, 0.3 mm vertical, and 0.4 mm longitudinal directions and average angular shifts of less than 0.3° in any rotational axis. All of these values are less than couch and couch‐mount precision.

**Table 1:** Average alignment accuracy for pediatric phantom.

		*Lateral (mm)*	*Vertical (mm)*	*Longitudinal (mm)*	*Pitch (°)*	*Roll (°)*	*Yaw (°)*
Initial Alignment Tests	Average	0.2	0.3	0.4	0.1	0.2	0.1
	Std. Dev.	0.2	0.2	0.3	0.1	0.1	0.1
Misalignment Test	Average	0.2	0.3	0.4	0.1	0.3	0.2
	Std. Dev.	0.2	0.2	0.3	0.1	0.1	0.1

The clinical patient positioning part of this study showed that the planar kV imaging alone is not adequate for accurate target positioning because it does not take into account the pitch and roll rotation required to align the treatment target. From our experience, we have found that relying only on orthogonal images for frameless SRS setups is not adequate for targeting. Our system predicted the pitch, roll, and yaw rotations required after orthogonal image alignment were as high as 4.5°, 1.6°, and 4.7°, respectively. Therefore, as a minimum, a volumetric imaging system (e.g., CBCT) is required to achieve accurate positioning of frameless stereotactic radiosurgery targets. The residual setup error results of our offline retrospective analysis of patients' target isocenters alignments using the 6 DOF system are shown in Figs. 4 and 5. The mean accuracy of the system in positioning patients' isocenters was less than 0.5 mm for any translational shifts and 0.3° for angular shifts around any axis of rotation (Table 2). The residual setup errors from the phantom study and patient data were within tolerance as stated by the American Association of Physicists in Medicine (AAPM) Task Group 54.^(22)^ These results provide confidence in our 6 DOF system to eliminate the need to obtain a third CBCT to verify setup before treatment delivery, reducing both patient radiation exposure and setup time.

**Table 2:** Average alignment errors for frameless targets treated using our 6 DOF system.

*Translational Setup Errors (mm)*	*Average*	*Std. Dev*
Lateral (LR)	0.4	0.3
Vertical (AP)	0.3	0.2
Longitudinal (SI)	0.5	0.3
*Rotational Setup Errors (degrees)*		
Pitch	0.3	0.2
Roll	0.3	0.3
Yaw	0.2	0.2

**Figure 6:**
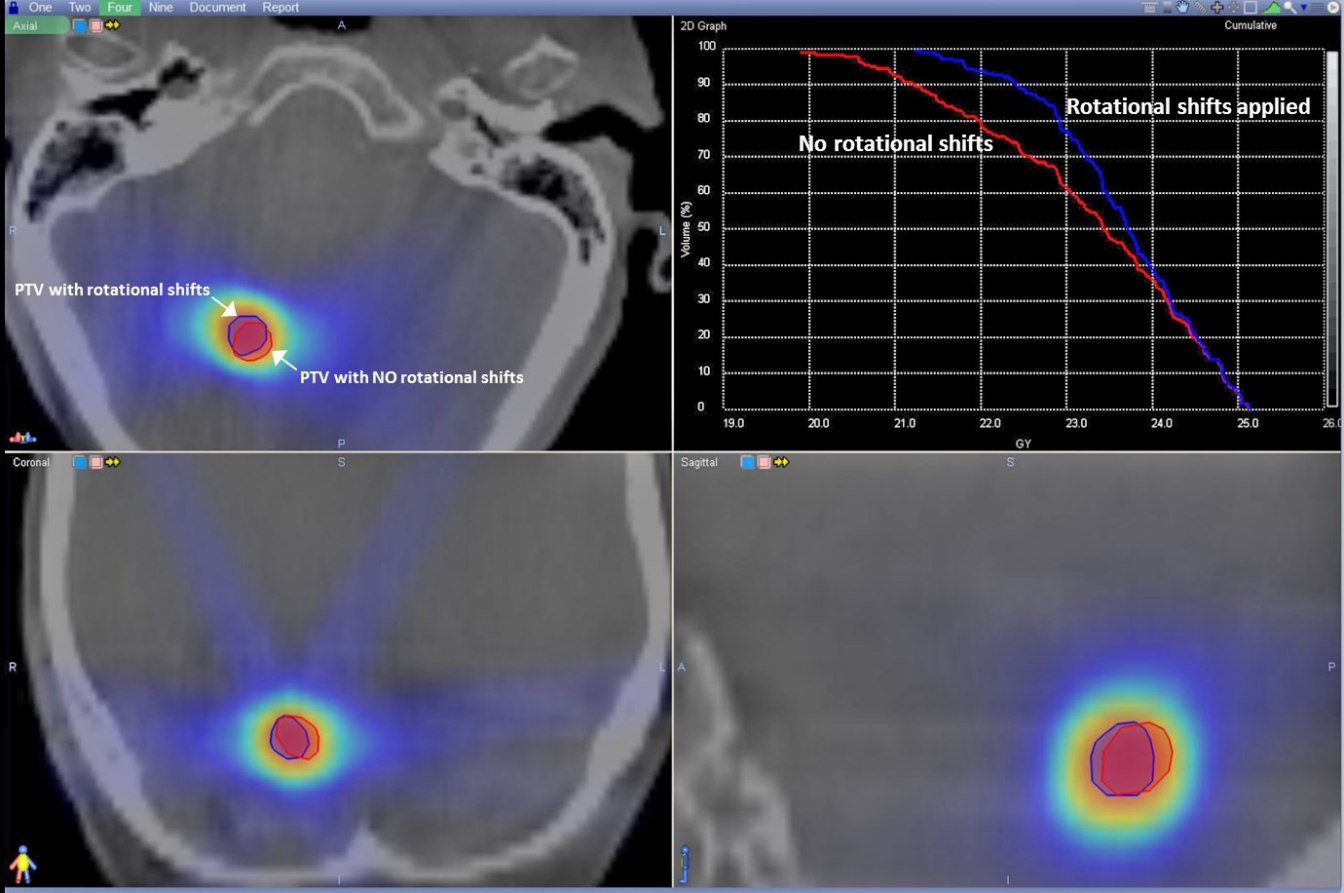
Comparison between 6 DOF and 4 DOF: the comparative DVH of a representative case shows the consequence of not applying pitch and roll corrections on dose cloud and PTV coverage.

To investigate the effect of not applying the rotational shifts, we calculated the dose cloud to the PTV with and without the roll and pitch rotational shifts and compared the results in several cases. A representative case, shown in Fig. 6, found an appreciable difference in the dose coverage, as observed from the DVHs, if the calculated rotations were not applied. This difference depended on the magnitude of the rotation, as well as shape and volume of the PTV. In some cases, the difference was modest; in other cases, a larger difference of up to 5% was observed.

## IV. DISCUSSION

As shown in Figs. 4 and 5, the maximum translational residual setup error was 1.2 mm and it was in the longitudinal (superior–inferior) direction. From the 63 isocenter positioning cases in this study, only six instances of translational error in one direction greater than 1 mm (all were < 1.2 mm) were observed. The majority of these misalignment errors (4 of 6 cases) were in the longitudinal direction, while the remaining two instances were in the lateral direction. The maximum residual setup error in the vertical direction was less than 0.9 mm. A previous study was conducted to examine patient stability during image‐guided frameless radiosurgery utilizing a thermoplastic mask similar to that used with our patients.^(9)^ In that report, intratreatment patient movement was assessed by comparing pre‐ and post‐treatment alignment. Differences of up to 1.0–1.2 mm were noted, with the greatest movement also in the longitudinal direction.^(9)^ We speculate that the higher incidence of translational errors <1 mm along the longitudinal axis could be attributed to the mask being less resistive in that direction. Only three cases with residual rotational setup error greater than 1° were noted, all involving yaw rotation. This could be attributed to a slight rotation of the patient head in the U‐frame mask between the time of the second and the third CBCT acquisitions. The mean accuracy of our 6 DOF system for target positioning in treated patients was less than 0.5 mm for any translational shifts and less than 0.3° for angular shifts around any axis of rotation, which is in line with our phantom study.

The alignment accuracy of frame‐based radiosurgery of 0.3 mm to 1.5 mm is reported by several studies.^9^, ^23^, ^25^ The AAPM Radiosurgery Task Group Report 54 states that the overall achievable positional uncertainty was 2.4 mm due to all error sources.^(22)^ These sources include variables such as target delineation inaccuracies, MR‐CT image registration inaccuracy, and position variation due to patient movements. As a comparison, the positioning error associated with stereotactic frame system was 1.4 mm. Many studies have also investigated the positioning accuracy of using bite plate and image‐guided frameless radiosurgery systems and reported positioning setup errors ranging from 0.4 to 1.2 mm.^9^, ^11^, ^12^, ^15^, ^26^

In our study, we found that the 6 DOF system provides an equivalent accuracy to previously investigated systems because of its ability to calculate and apply three translational — as well as rotational — shifts. Our 6 DOF system is a unique system compared to other commercially available systems^13^, ^29^, ^30^ because it uses volumetric imaging CBCT to obtain an accurate target placement, whereas other systems use planar X‐ray images for targeting. A unique feature of our 6 DOF system versus other 6 DOF systems is that those systems compare DRRs with planar X‐ray images, whereas our 6 DOF system relies on volumetric image sets by registering live CBCT to PCT. Studies show that DRRs and X‐ray images are not optimal for image registration due to substantial overlap of structures.^13^, ^31^ However, this is not an issue with CBCT and PCT registration because 3D registration algorithm of CBCT to PCT provides better registration accuracy.^13^, ^16^, ^31^

Other investigators have reported similar findings if 6 DOF is not used and only 4 DOF were applied. They too reported a loss of dose coverage of up to 5% to the PTV if all three rotational shifts were not applied.^(32)^ In another study, the effect of applying 4 DOF not 6 DOF shifts was investigated when treating stereotactic body radiation therapy (SBRT) cases, such as spine radiosurgery.^(33)^ The authors reported significant decrease in the PTV dose coverage for spinal radiosurgery if additional rotational shifts were not taken into consideration. These authors also reported that the shape and size of PTV play an important role in loss of dose coverage. They showed that failure to apply even medium range rotational shifts ($\text{roll}=1.65^{{}^{\circ}}$ and $\text{pitch}=1.23^{{}^{\circ}}$) will lead to a significant loss of dose coverage for highly irregular tumors from 57.2% to 49.1% at 10 Gy dose level.^(33)^ Based on these publications and our own work, it is clear that only adjusting for 4 DOF may, in some cases, lead to significant compromise in PTV coverage. While it may not be necessary to make the pitch and roll adjustments in all cases, it would be difficult to determine, in a timely fashion while the patient is on the treatment table, whether a particular rotational adjustment would make a significant difference in PTV coverage. Since performing the additional match with 6 DOF in our registration system only adds a relatively short amount of time to the overall process, we advocate making the precise match in all cases.

Although frameless radiosurgery systems utilizing a bite plate are reasonably accurate,^11^, ^12^, ^28^ they have limitations for use in pediatrics because of the need for a cooperative subject, and in elderly patients who often lack maxillary teeth.^(28)^ Since our 6 DOF system without a bite plate provides equivalent or better accuracy than optically guided bite plate‐based systems, it is a very attractive option for patients, especially the elderly and children. Additional advantages of this frameless system are the ability to reproduce and deliver stereotactic fractionated radiosurgery, and to allow greater sparing of critical structures such as brainstem and optic chiasm.^34^, ^36^ Such fractionated stereotactic treatment is also suitable for large lesions to avoid delivering high doses in a single fraction to a large volume of brain tissue.^(8)^

## V. CONCLUSIONS

Accurate and efficient frameless radiosurgery positioning without the use of bite plate optical guidance is achievable using our 6 DOF registration method. Our system provides submillimeter positioning accuracy, which is well within the guidelines for radiosurgery systems as stated by the AAPM Radiosurgery Task Group Report 54. We recommend applying both spin and pitch rotational correction in all cases to avoid compromising coverage to the PTV and to deliver the prescribed dose. This system is relatively inexpensive compared to a commercially available 6 DOF system, minimizes patient discomfort, and allows for the treatment of pediatric patients without the need for anesthesia, as well as elderly patients who often have dental issues.
